# LC-QTOF-MS Chemical Characterization, Cytotoxicity, and Antimicrobial Activity of Blue Geopropolis: An Emerging Type of Brazilian Propolis

**DOI:** 10.3390/ph19081256

**Published:** 2026-08-10

**Authors:** Vitor Luis Fagundes, Bruno Fernandes de Oliveira, Renato Rau, Luana Mota Ferreira, Francinete Ramos Campos, Monica Surek, Roberto Pontarolo

**Affiliations:** 1 Programa de Pós-Graduação em Ciências Farmacêuticas, Universidade Federal do Paraná, Curitiba 80210-170, Paraná, Brazil; vitorluisfagundes@ufpr.br (V.L.F.); fernandesoliveira@ufpr.br (B.F.d.O.); luanamota@ufpr.br (L.M.F.); 2 Instituto de Tecnologia do Paraná (TECPAR), Curitiba 81350-010, Paraná, Brazil; rrau@tecpar.br; 3 Embrapa Semiárido, Rodovia BR 428, Km 152, Zona Rural, Petrolina 56334-899, Pernambuco, Brazil; monicasurek13@gmail.com

**Keywords:** propolis, natural products, phenolic compounds, stingless bee propolis, geopropolis

## Abstract

**Background/Objectives**: This study characterized blue geopropolis from *Melipona quadrifasciata quadrifasciata* (southern Brazil) by comparing its chemical composition, cytotoxicity, antimicrobial activity, and coloration mechanisms directly against green and red *Apis mellifera* propolis under identical experimental conditions. **Methods**: Ethanolic extracts of blue geopropolis, green, and red propolis were prepared, followed by hexane and chloroform fractionation. Chemical composition was analyzed by LC-qTOF-MS. Cytotoxicity was assessed in L929 fibroblasts, and antimicrobial activity was determined by minimum inhibitory concentration (MIC) assays against bacterial and fungal strains. The blue coloration was investigated using NaOH and FeCl_3_ treatments to evaluate the possible involvement of phenolic–iron complexes. **Results**: Fifty-nine compounds were identified across the three samples, revealing several shared phenolic constituents as well as distinctive chemical features. None of the extracts showed activity against Gram-negative bacteria, including *Escherichia coli* and *Pseudomonas aeruginosa* (MIC > 1000 µg/mL). In contrast, activity was observed against Gram-positive bacteria, with MIC values ranging from 20.8 to 1000 µg/mL. Among the samples tested, blue geopropolis showed the strongest activity against Gram-positive bacteria. Blue geopropolis exhibited no significant activity against *Candida albicans* or *Candida parapsilosis* (MFC > 1000 µg/mL) but showed variable activity against *Sporothrix* spp. The colorimetric assay indicated that phenolic–iron complexation may contribute to the blue color. **Conclusions**: Despite its distinct entomological origin, blue geopropolis shared chemical traits with green and red propolis while demonstrating superior Gram-positive antibacterial activity, a higher selectivity index, and potential antifungal applications against *Sporothrix* spp., highlighting its promise for biotechnological and pharmacological use.

## 1. Introduction

Current global health challenges, such as the emergence of new diseases and the rise in drug resistance, highlight the need for new, more effective therapeutic agents. However, this process involves long research periods and high costs. In this context, natural products have emerged as promising sources for identifying and isolating compounds with therapeutic potential [1,2]. Their therapeutic efficacy is widely supported by scientific evidence and historical use and is often associated with the high complexity of natural product extracts, which favors synergistic effects and simultaneous actions [3].

One bioproduct that has aroused great interest in the scientific community is propolis. It is a resinous substance of complex nature, produced by bees from the collection of plant exudates present in flower buds, flowers, and leaves, which are subsequently modified by the addition of bee secretions and wax [4]. The empirical use of propolis as a therapeutic agent dates back to antiquity and is widely documented across different civilizations [5]. Its medicinal properties were described by scholars of Greco-Roman medicine, including Aristotle, Pliny the Elder, Galen, Cornelius Celsus, and Dioscorides. It is also noteworthy that Hippocrates (460 BC–377 BC) was one of the first to report the application of this substance in the treatment of wounds and ulcers [6].

Although primarily known for its healing properties and antibacterial activity, propolis is a multifunctional biological matrix with a broad spectrum of pharmacological activities applicable to health promotion strategies. In vivo and in vitro studies have demonstrated that various symptoms associated with different pathologies can be attenuated with propolis [7]. Currently, there is growing interest in investigating its therapeutic properties, with evidence supporting its potential application in the medical and pharmaceutical fields. Among its main biological activities, the antibacterial, antiviral, anti-inflammatory, antioxidant, immunomodulatory, and antiproliferative effects stand out [8,9]. Due to its broad spectrum of action, propolis has potential as an adjuvant in the prevention and treatment of various conditions, including diabetes, infections, allergies, hypertension, obesity, and cancer [10,11].

The properties of propolis result from its complex and highly variable chemical composition. To date, more than 1000 compounds have been identified, belonging mainly to the classes of alkaloids, terpenes, flavonoids, phenolic acids, aliphatic acids, carbohydrates, vitamins, and minerals [12,13]. This composition is influenced by factors such as the seasonality of collection, the flora available to the bees in the region, and the genetic variability of the producing bees [14,15]. Due to this compositional heterogeneity, there are different types of propolis in nature, which can be classified based on their physicochemical properties, including color, texture, and chemical profile. In this context, a wide range of chromatic variation is observed, from yellowish-brown, greenish-brown, and reddish-brown tones to dark red [15].

In Brazil, 14 types of propolis have been described, classified by botanical origin, physicochemical properties, and geographic location [14]. Additionally, some species of stingless bees (Meliponini) produce a different type of propolis, in which soil particles (earth or sand) are incorporated into the resinous matrix; this material is called geopropolis [16]. Among the most recently described bee products is a blue-colored geopropolis (blue propolis) produced by *Melipona quadrifasciata quadrifasciata*, found on the southern coast of Brazil. Its name is associated with its bluish color, and, to date, there are no records of its occurrence in other regions of the world.

Although geopropolis differs from conventional propolis produced by *Apis mellifera* in its entomological origin and because it incorporates soil particles into the resinous matrix, comparative studies remain scientifically relevant. Such comparisons allow the identification of shared and distinctive chemical constituents, contribute to understanding the relationship between chemical composition and biological activity, and help assess the pharmacological potential of bee products obtained from different species under the same analytical conditions.

Despite the consolidated knowledge of propolis’s general properties, blue geopropolis still lacks a comprehensive characterization of its physicochemical composition and biological activities. In this context, the present study aimed to characterize the blue geopropolis produced by *Melipona quadrifasciata quadrifasciata*, collected on the coast of southern Brazil, through the evaluation of its chemical composition, cytotoxicity, and antimicrobial activity. These characteristics were comparatively evaluated with those of green and red propolis produced by *Apis mellifera*, using identical analytical and biological approaches to identify similarities and differences in their chemical profiles and biological properties, rather than to establish equivalence between these bee products. Additionally, the study sought to investigate the possible chemical basis and mechanisms underlying the distinctive blue coloration of this geopropolis.

## 2. Results and Discussion

To date, there are no reports in the scientific literature describing the chemical composition and biological activities of blue geopropolis, making this, to the best of our knowledge, the first comprehensive characterization of this bee product. In the absence of previous studies, green and red propolis produced by *Apis mellifera*, whose chemical composition and biological properties have been extensively characterized, were included as reference materials for comparative analyses. Such comparisons were intended to identify similarities and differences in chemical composition and biological activity, thereby providing a framework for assessing the pharmacological potential of blue geopropolis rather than establishing equivalence among products of distinct entomological origin.

### 2.1. Obtaining Propolis Extracts

Three propolis extracts were obtained, named blue propolis ethanolic extract (BPEE), green propolis ethanolic extract (GPEE), and red propolis ethanolic extract (RPEE). In addition, hexane and chloroform fractions were obtained from each extract, designated as BPEE-H and BPEE-C, GPEE-H and GPEE-C, and RPEE-H and RPEE-C, respectively. In total, nine propolis extracts were obtained, including ethanolic extracts and their respective fractions, which were subsequently used for biological assays and chemical characterization.

For blue propolis, methanol/water partitioning did not yield detectable compounds, possibly due to their low affinity for the hydroalcoholic phase. In the subsequent step, the constituents were predominantly solubilized in the chloroform fraction, leaving no compounds susceptible to extraction with ethyl acetate, making it impossible to obtain this fraction for this sample. Although ethyl acetate fractions were obtained for red and green propolis, they were disregarded to maintain methodological standardization among the samples.

The distribution of the obtained fractions indicates a predominance of low- to medium-polarity compounds, evidenced by greater recovery of material in the hexane and chloroform fractions and by the absence of significant extraction in the methanol/water and ethyl acetate steps. These results suggest a predominantly nonpolar chemical profile for blue propolis.

In general, the chemical compounds present in propolis samples are extracted with different solvents, the most common being ethanol, water, hexane, ethyl acetate, and chloroform [17]. Ethanol extraction is one of the most traditional and simple methods for obtaining tinctures for curative purposes [18]. As part of the extraction process, fractionation of crude extracts to investigate biological activities is a fundamental strategy for identifying which compounds are responsible for the observed activity, using solvents of different polarities. Furthermore, this approach enables evaluation of potential interactions among the constituents, such as synergistic or antagonistic effects, which can influence the biological response [19]. In this way, some fractions may exhibit greater biological activity than the crude extract, thereby increasing its therapeutic value [20].

### 2.2. Chemical Characterization

Blue propolis was collected in the coastal region of Paraná, Brazil, and, to the best of our knowledge, this is the first study to investigate the major compounds present in this new type of propolis. Considering the high chemical complexity of propolis extracts, liquid chromatography coupled with mass spectrometry (LC-MS) was used to characterize the constituents.

In this study, UPLC-ESI-qTOF analysis in both positive and negative modes revealed a complex mixture of compounds, with identified constituents presented in Table 1. In general, the samples showed similar qualitative profiles, with the main difference being the relative abundance of the compounds. In total, 59 compounds were identified in the propolis extracts; of these, 42 are present in blue propolis, 50 in green, and 51 in red. Identification was performed by comparing the exact mass (error ≤ 5 ppm) and fragmentation spectra with data available in the literature [21,22,23,24,25,26,27,28,29,30,31,32]. The compounds identified here belong mainly to the phenolic classes (including flavonoids, phenolic acids, phenolic lipids, and prenylated cinnamic acids), as well as terpenoids, such as diterpenes and triterpenes.

In general, propolis is a complex matrix composed of a wide variety of bioactive molecules, mostly derived from primary and secondary plant metabolites [33]. Primary metabolites, such as amino acids, organic acids, and carbohydrates, are essential for the maintenance of life and act as precursors of secondary metabolites. These, in turn, play a fundamental role in plant interactions with the environment. Secondary metabolites can be grouped into three main chemical classes: phenolic compounds, terpenoids, and alkaloids [34]. In propolis, phenolic acids and flavonoids stand out as major constituents, in addition to other classes of compounds, such as ketones, aldehydes, chalcones, dihydrochalcones, terpenoids, amino acids, aliphatic and aromatic acids, esters, carbohydrates, vitamins, and metals [35].

Flavonoids were the most representative class, with 31 distinct compounds identified. Blue propolis presented the lowest number of detected flavonoids (*n* = 14), followed by green propolis (*n* = 21) and red propolis (*n* = 25). Among these, compounds 3, 4, 12, and 24 were exclusive to red propolis, while 10 was identified exclusively in green propolis. Flavonoids play an important role among the active substances in the effects of propolis [36]. The different colors of propolis found in nature are attributed to flavonoids originating from different parts of the plant [37].

Three compounds classified as phenolic acids were also identified: 1 (exclusive to green propolis), 19, and 39. These compounds are known as valuable antioxidants, anticancer, antidiabetic, anti-inflammatory, and antibacterial agents [38].

In addition to flavonoids and phenolic acids, other phenolic subclasses were identified in the propolis samples. Six phenolic lipids were detected, including anacardic acid derivatives (compounds 34, 35, 37, and 38), an unidentified anacardic acid (compound 41), and xanthochymol (guttiferone E; compound 43). These compounds are relevant because phenolic lipids have been associated with antioxidant, cytotoxic, and antimicrobial effects, which may contribute to the biological activities observed for propolis extracts [38]. Eight prenylated cinnamic acids were also identified, including compounds 2, 23, 29, 33, 36, 40, 42, and 44. Notably, compound 33 was detected only in green propolis. Together, these findings reinforce the chemical diversity of the extracts and indicate that non-flavonoid phenolic constituents may also play an important role in their bioactive profile.

Terpenoids belonging to the diterpene and triterpene classes were detected. Among the diterpenes, seven distinct compounds were identified, five of which were observed exclusively in blue propolis (Compounds 45, 47, 49, 50, and 51). However, these compounds have been previously described in other types of propolis, indicating that they cannot be considered exclusive markers of blue propolis. Additionally, three triterpenes were identified and present in all three types of propolis analyzed. Lipids (Compounds 55, 56, and 57) were also detected in all types of propolis.

Unlike what was observed for other classes of compounds, terpenoids did not show a clear separation between the fractions. All compounds identified in the ethanolic extract were also detected in the hexane and chloroform fractions, indicating low selectivity of the fractionation process for this class. This behavior can be explained by the structural nature of the identified terpenoids, particularly the acidic diterpenes, which have intermediate polarity and therefore exhibit affinity for various organic solvents [39]. In addition, the complexity of the propolis matrix favors co-extraction of compounds, leading to overlap between fractions. These results suggest that, for terpenoids, the employed fractionation did not promote significant selective enrichment. The selectivity of the extraction step is determined by the concentration of the target compounds in the extraction phase relative to the matrix, and, to be useful, the recovery of the target compounds must be adequate [40]. Therefore, the same compound can be extracted in one phase for one extract and in another phase for a different extract, since the selectivity of the extraction also depends on the matrix in which the substance is embedded.

For phenolic compounds, a consistent distribution pattern was not observed between the extracts and their respective fractions. In some cases, certain compounds were detected in all fractions of one type of propolis, whereas in another type, although present in the ethanolic extract, these same compounds were not detected in the corresponding fractions. This behavior indicates that the distribution of compounds is influenced not only by their intrinsic physicochemical properties but also by interactions with the sample matrix. Matrix effects may alter analytical responses by suppressing or enhancing signals, potentially affecting compound detection and, in some cases, leading to false-negative results [41]. In addition, variations in metabolite concentrations, partitioning behavior during fractionation, and analytical limitations may further affect compound detection [42]. Importantly, the absence of detection should not be interpreted as definitive evidence of compound absence. Instead, it may reflect incomplete recovery during extraction or fractionation, low metabolite abundance, matrix-related signal suppression or enhancement, or limitations of the analytical method employed. Therefore, these results should be interpreted with caution and reinforce the need for complementary analytical strategies to better understand the chemical distribution of phenolic constituents in propolis extracts.

Fractionation of extracts is the first step in “bioassay-guided fractionation” and can help determine the extract’s activity, with two possible outcomes after fractionation: maintenance, increase, or reduction of activity [43]. When there is a reduction in activity in one of the fractions relative to the total extract, this means that the action is due to a synergistic interaction between the constituents of the total extract. When maintenance or an increase in activity is observed, this indicates that the action is due to isolated compound(s) [44]. Thus, although the differences in the chemical composition of the extracts and their fractions are not evident, these findings highlight the chemical complexity of propolis and underscore the need for more targeted approaches, such as the isolation of compounds or controlled combinations, to elucidate the mechanisms of interaction among metabolites.

Mass spectrometry is one of the main techniques used in the analytical investigation of complex molecules and mixtures. High-resolution mass spectrometry (HRMS), in particular, has become an essential tool in metabolomics studies, allowing the detection of a wide range of analytes with high resolution, mass accuracy, molecular specificity, and sensitivity across broad mass ranges. In this context, the inherent complexity of natural product matrices demands the use of advanced analytical tools for the adequate characterization of the compounds present [45]. Metabolomics has expanded significantly in the study of natural products, driven by interest in understanding the metabolic profiles of complex biological systems. In this scenario, mass spectrometry stands out as the primary data-acquisition technique due to its high sensitivity and large-scale analysis capabilities [46].

Despite these challenges, LC-MS remains one of the most powerful and widely used platforms for untargeted metabolomics due to its high sensitivity, broad metabolite coverage, and capability to detect compounds across a wide range of chemical classes. In untargeted approaches, the structural annotation of unknown metabolites remains a major challenge because many detected features have not been previously characterized [45]. Data processing and interpretation require advanced bioinformatic and multivariate statistical tools to distinguish biologically relevant signals from analytical artifacts. In addition, a single metabolite may generate multiple ion species, including adducts, isotopes, and in-source fragments, which must be carefully considered during data analysis. Differences in ionization efficiency among analytes can also influence signal intensity and metabolite coverage, particularly for low-abundance compounds [47]. Nevertheless, the use of quality-control procedures, optimized workflows, and MS/MS-based annotation strategies substantially improves the reliability and robustness of LC-MS metabolomics studies.

The chemical characterization obtained in this study should be considered tentative, as compound annotation was based on accurate mass measurements, fragmentation patterns, and comparison with literature data. The use of authentic analytical standards could increase the confidence of identification by enabling direct comparison of retention times, accurate masses, and MS/MS spectra [48]. However, this strategy is limited by the commercial availability and cost of standards, as well as by the high chemical complexity of propolis extracts. Therefore, the structural confirmation of some constituents, especially those not fully described in the literature or spectral databases, may require complementary analytical approaches in future studies. These limitations help explain why some compounds detected in the extracts could not be unequivocally identified.

### 2.3. Evaluation of Cellular Cytotoxicity

**Figure 1:**
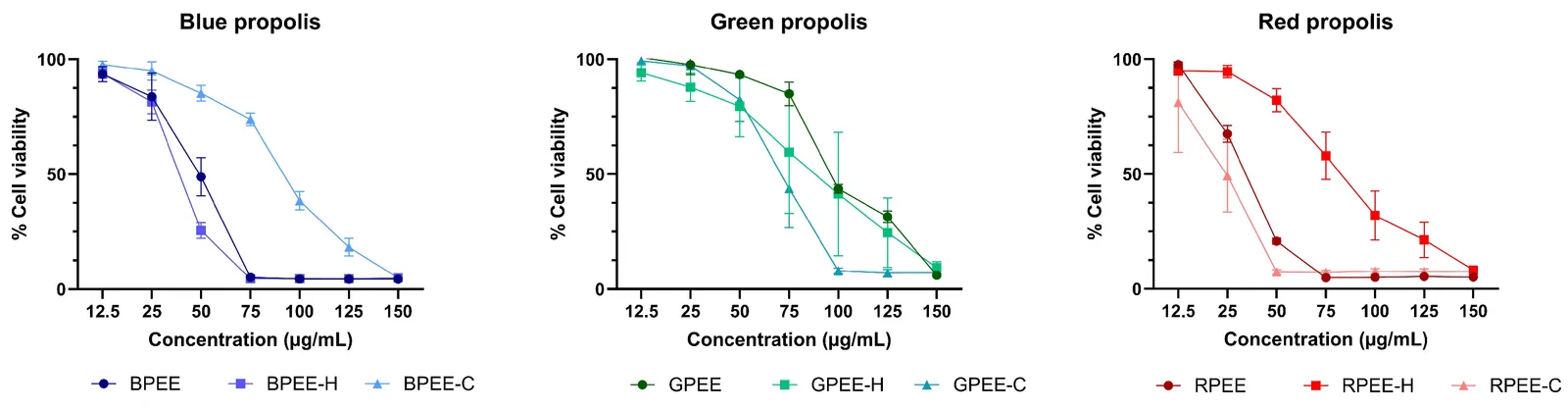
Cytotoxicity of different propolis extracts when tested against the L929 cell line.

The cytotoxic effects of propolis extracts (blue, green, red) were evaluated using the L929 cell line (non-tumor mammalian cells), a cell line commonly used to assess the cytotoxicity of natural products and biomaterials and recommended by international standards for its sensitivity [49]. In this study, cytotoxicity evaluation demonstrated that all propolis extracts reduced the viability of the L929 cell line in a concentration-dependent manner (Figure 1).

The RPEE, RPEE-C, and BPEE-H extracts demonstrated greater toxicity with IC_50_ values (Table 2) of 29.8 ± 1.3 (mean ± standard deviation), 25 ± 0.6, and 39 ± 2, respectively. Meanwhile, the GPEE-H extract showed lower cytotoxicity, with an IC_50_ of 138 ± 17 μg/mL. Among the ethanolic extracts, GPEE proved to be less cytotoxic, while there was no significant difference between the cytotoxicity of the BPEE and RPEE extracts.

In one study, it was demonstrated that different propolis samples produced in Colombia exhibit IC_50_ values in the L929 cell line ranging from 320.6 to 4341.0 μg/mL [50]. Red propolis did not demonstrate cytotoxicity in Vero and LLC-PK1 cell lines at concentrations up to 100 μg/mL, while exhibiting moderate cytotoxicity against tumor cells [51]. Another study evaluated the cytotoxicity of a combination of 3 types of propolis (American, Asian, and European) in 5 cell lines (MG63, L929, HL60, THP-1, and human mesenchymal cells). It was found that the IC_50_ values for the L929 cell line ranged from 17.9 to 18.6 µg/mL. This value was lower than that of the other cell lines, suggesting greater susceptibility of fibroblasts (L929) to the toxicity of preparations containing propolis extracts [49]. Evaluation of cytotoxicity in cell lines can vary depending on cell sensitivity and other factors, such as the number of passages the cell line has undergone. Therefore, comparisons of data from the literature may vary due to cell-to-cell differences, even when they are from the same cell line.

In general, extracts can be considered cytotoxic if the IC_50_ value is less than 20 μg/mL after 48 or 72 h of incubation; cytotoxic activity is considered moderate with values between 30 and 100 μg/mL, and weak for concentrations above 100 μg/mL for crude extracts [52]. Therefore, extracts with IC_50_ values below 100 μg/mL can be considered promising sources for the development of new anticancer drugs [53]. The cytotoxic effect is of interest when targeting tumor cells; the results of the present study demonstrate that propolis extracts affect cell viability. This effect can be explored in the control of tumor cells; therefore, it would be interesting to evaluate the selectivity index of propolis extracts’ cytotoxicity in non-tumor cells versus tumor cells, assessing their specificity in targeting target cells. Therefore, it is necessary to continue evaluating the cytotoxic profile of propolis and its active compounds to establish analytical standards [50]. In this way, by evaluating the selectivity of propolis extracts in tumor cells, propolis can be explored as an anticancer agent, as it has been shown that its bioactive compounds inhibit cancer progression by targeting multiple signaling pathways [54].

### 2.4. Evaluation of Antibacterial Activity

The antibacterial activity of propolis extracts was evaluated against Gram-positive and Gram-negative bacteria, and MIC and MBC were determined using the broth microdilution method. No activity (MIC > 1 000 μg/mL) was observed against Gram-negative bacteria (*Escherichia coli* and *Pseudomonas aeruginosa*). On the other hand, the extracts showed different levels of activity against Gram-positive bacteria. It is generally considered that propolis has more pronounced antibacterial activity against Gram-positive bacteria than against Gram-negative bacteria; the lower activity against Gram-negative bacteria is attributed to the structure of the outer membrane and the production of hydrolytic enzymes by these bacteria [55]. These enzymes can compromise the activity of the active ingredients in propolis [4]. Gram-negative bacteria have a thin peptidoglycan layer, while Gram-positive bacteria have a thicker layer. It is understood that one of the bacterial components affected by propolis must be the peptidoglycan layer, and the outer membrane of Gram-negative bacteria limits the passage of propolis components and prevents their action on the thin peptidoglycan layer [20].

The effect of propolis extracts against strains of *Staphylococcus* spp. and *Enterococcus* spp. was evaluated. Strains of *Staphylococcus aureus* and *Enterococcus faecium* are part of the ESKAPE group, a priority group designated by the WHO due to their accelerated resistance, comprising *E. faecium*, *S. aureus*, *Klebsiella pneumoniae*, *Acinetobacter baumannii*, *P. aeruginosa*, and *Enterobacter* spp. [56]. Thus, new therapies for controlling these pathogens are urgently needed, as are resources to bring these agents to developing countries, where resistance is increasing more rapidly [57].

**Figure 2:**
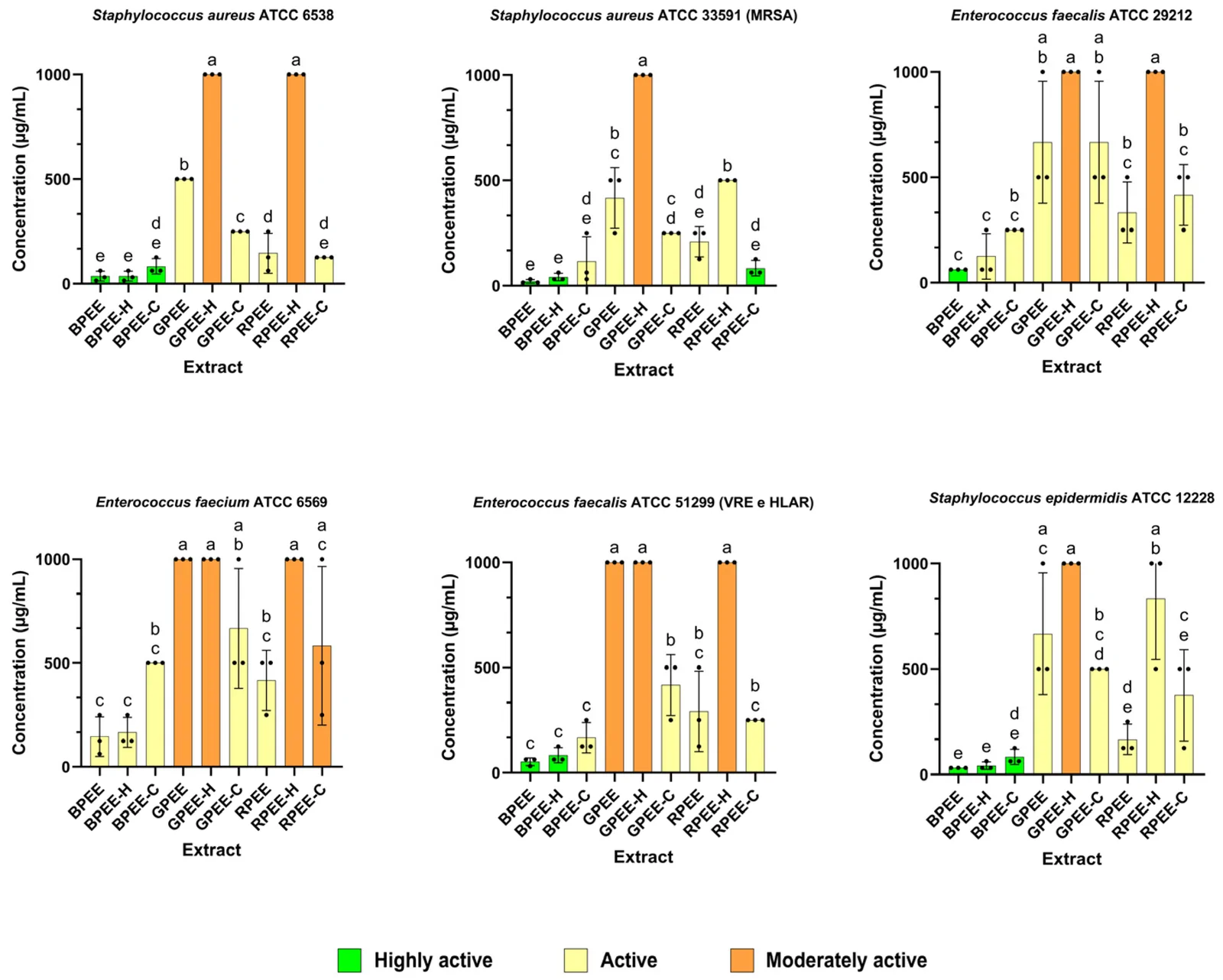
Graphical representation of MIC values of propolis extracts. Ethanolic Extract of Blue Propolis (BPEE), Ethanolic Extract of Blue Propolis-Hexane Fraction (BPEE-H), Ethanolic Extract of Blue Propolis-Chloroform Fraction (BPEE-C), Ethanolic Extract of Green Propolis (GPEE), Ethanolic Extract of Green Propolis-Hexane Fraction (GPEE-H), Ethanolic Extract of Green Propolis-Chloroform Fraction (GPEE-C), Ethanolic Extract of Red Propolis (RPEE), Ethanolic Extract of Red Propolis-Hexane Fraction (RPEE-H), Ethanolic Extract of Red Propolis-Chloroform Fraction (RPEE-C). Statistical differences were evaluated by one-way ANOVA followed by Tukey’s post hoc test. ^abcde^ Bars sharing the same letter are not significantly different (*p* > 0.05).

The MIC of the extracts for Gram-positive bacteria ranged from 20.8 to 1000 μg/mL (Table 3 and Table 4) (Figure 2). The antimicrobial activity of plant extracts can be interpreted using minimum inhibitory concentration (MIC) values. Values < 100 μg/mL are considered highly active; 100–500 μg/mL, active; 500–1000 μg/mL, moderately active; 1000–2000 μg/mL, low activity; and >2000 μg/mL, inactive. In general, phytochemical products with MICs between 100 and 1000 μg/mL are considered to have antimicrobial activity [58]. The MIC values obtained for the positive controls were within the expected range, confirming the reliability of the susceptibility assays.

The MBC/MIC ratio was used to assess the antibacterial behavior of the propolis extracts against *Staphylococcus* spp. All extracts and fractions obtained from blue propolis exhibited MBC/MIC ratios greater than 4, suggesting a predominantly bacteriostatic effect. In contrast, all green propolis-derived samples presented MBC/MIC ratios below 4, indicating predominantly bactericidal activity. For red propolis, both bactericidal and bacteriostatic profiles were observed, with MBC/MIC ratios varying above and below the threshold value of 4. Against *Enterococcus* spp., a similar trend was observed, although the chloroform fraction of blue propolis (BPEE-C) exhibited MBC/MIC ratios below 4, suggesting bactericidal activity. All green propolis extracts showed MBC/MIC ratios below 4, whereas most red propolis samples also exhibited values indicative of bactericidal action. In some cases, involving green and red propolis, the MBC/MIC ratio could not be determined because the MBC value was equal to or greater than 1000 µg/mL, corresponding to the highest concentration tested.

The selectivity index was calculated to evaluate the relationship between antibacterial activity and cytotoxicity. Against *Staphylococcus* spp., only blue propolis-derived extracts exhibited SI values greater than 1, indicating a more favorable balance between antibacterial efficacy and cytotoxicity. For *Enterococcus* spp., SI values were generally lower than 1 for all samples, except for the chloroform fraction of blue propolis (BPEE-C), which showed an SI of 2.33, suggesting selective antibacterial activity against this bacterial group.

It is observed that only extracts derived from blue propolis (BPEE, BPEE-C, BPEE-H) exhibit activity classified as “highly active,” except for the RPEE-H extract, which also shows this classification when tested against *S. aureus* (MRSA). There is a significant difference in antibacterial activity among the propolis extracts, with blue propolis exhibiting the strongest activity. In this sense, the activity of the chloroform and hexane fractions is lower than that of the ethanolic extract, demonstrating that the effect observed with the BPEE extract may be due to a synergistic action between its constituents; when these constituents are separated by partitioning, the effect is reduced. The same effect is not observed in extracts derived from green and red propolis, suggesting that the observed action is due to isolated compounds rather than synergistic effects. This effect has already been demonstrated in another study, which evaluated the biological activity of red propolis and found no synergistic action [59].

Based on the selectivity index, blue propolis shows the best indices, indicating potential for future applications. Selectivity indices >1 indicate lower cytotoxicity against mammalian cells (L929) compared to microorganisms. Consequently, compounds with higher SI have a safer profile [22]. The study of cytotoxicity in other non-tumor cell lines can better elucidate the cytotoxicity of propolis extracts and even improve the selectivity index, since it has already been shown that the L929 cell line appears to be more sensitive [49]. One study concluded that Polish propolis exhibits considerable anti-staphylococcal activity, with MIC and MBC values for *Staphylococcus epidermidis* (ATCC 12228) ranging from 32 to 512 µg/mL. For two strains of *S. aureus* (ATCC 25923, ATCC 29213), the range was 128–512 µg/mL [60].

One study evaluated the antimicrobial activity of ethanolic extracts of different types of Brazilian propolis (yellow, green, brown and red), in which it was found that yellow and brown propolis showed weak action against the microorganisms tested and the extracts of green and red propolis showed high antibacterial activity against gram-positive bacteria, particularly green propolis against *S. aureus* (MIC = 159 μg/mL), *Enterococcus faecalis* (MIC = 310 μg/mL) and MRSA (MIC = 630 μg/mL) [61]. Extracts prepared with propolis collected in different regions of Brazil demonstrate activity against *S. pneumoniae* and *S. aureus*, with MIC ranges between 0.2–0.8 μg/mL and 1.6–52.4 μg/mL, respectively; in this study, activity against *K. pneumoniae* was also evaluated, but all extracts proved inactive against this microorganism [62]. A study evaluated the activity of Portuguese propolis against strains isolated from the clinical environment and reported MIC values of 8.0 mg/mL for *E. faecium* and 1.5 mg/mL for *E. faecalis* [63].

However, it is observed that there is a variation in the MIC values reported in the literature between the same types of propolis and between different types. This is due to variations in their composition, geographical location, extraction methods, collection practices, time and season of collection, and age of the propolis [64]. There is no consensus or standard in the literature on procedures for preparing propolis extracts (solvents, concentrations, and extraction times used) and for evaluating the antimicrobial activity of propolis (concentrations evaluated and methods used). For example, the extraction method affects the antimicrobial properties of the extract, the yield, and the content of phenolic compounds and flavonoids [65]. Some studies use qualitative methods to evaluate the antimicrobial activity of propolis, while others use quantitative methods, such as macro- and microbroth dilution. It is known that quantitative methods present more consistent results [66]. Therefore, comparing the biological activity of propolis with data from the literature is limited, as the conditions used differ. Thus, variations in composition, the extraction method used, and the method chosen to evaluate antimicrobial activity contribute to the inconsistencies and contradictions in the literature.

### 2.5. Evaluation of Antifungal Activity

The antagonistic action of propolis extracts against *Candida* spp. and *Pichia kudriavzevii* was evaluated (Table 5), with the results expressed as minimum fungicidal concentration (MFC). The extracts derived from blue propolis did not show activity against *Candida albicans* and *Candida parapsilosis* strains (MFC > 1000 μg/mL).

For *C. albicans*, antagonistic activity was observed with the action of green and red propolis, with the RPEE fraction showing more pronounced activity. For *C. parapsilosis*, the results presented by the hexane fractions (GPEE-H and RPEE-H) of green and red propolis were similar. The BPEE and BPEE-C extracts did not show activity against *P. kudriavzevii*. For this microorganism, the hexane fractions of green and red propolis (GPEE-H and RPEE-H) also showed greater antifungal activity.

The results suggest that the antifungal activity of green propolis does not primarily result from a synergistic effect among the constituents of the ethanolic extract, since the chloroform and hexane fractions showed greater biological activity than the total extract. This finding indicates that compounds with greater affinity for low-polarity solvents may be the main agents responsible for the observed activity, and that fractionation contributed to the concentration of these bioactive metabolites.

For red propolis, a distinct behavioral pattern is observed that depends on the fungal species evaluated. Against *C. albicans*, the crude extract (RPEE) showed greater antifungal activity, suggesting that the inhibitory effect may be associated with synergistic interactions between different chemical constituents present in the complex matrix of the extract. In contrast, against *I. orientalis*, the hexane fraction demonstrated greater activity, indicating that, in this case, less polar compounds may play a predominant role in the antifungal activity.

Taken together, these results show that the inhibitory activity of propolis can be modulated by both the chemical composition of the fractions and the intrinsic susceptibility of the fungal species evaluated, suggesting that different groups of metabolites may act through distinct mechanisms of action depending on the target microorganism.

The use of propolis in *Candida* spp. strains is an interesting strategy, since it has already been verified that propolis does not induce resistance in strains and can act on strains already resistant to conventional antifungals [67]. *Candida* spp. species are the main ones studied in susceptibility tests. The use of other microorganisms in these assays would be interesting, as some are common skin pathogens, such as *Trichosporon* spp. and *Malassezia* spp., which are poorly studied [62].

As in another study, the concentrations of propolis extracts required to inhibit the growth of *Candida* spp. and *I. orientalis* are relatively high, exceeding the IC50 values in non-tumor cells [63]. The use of these propolis is not recommended for the treatment of *Candida* spp. and I. orientalis infections except for GPEE-H, which has an IS close to 1.40 for *I. orientalis*. Although there is promising data in the literature demonstrating the action of propolis against *Candida* spp. strains, the same effect was not found in the present study. This may be due to variations in propolis composition, as it has been shown that the action against *Candida* spp. strains varies in rainy and dry seasons, with the effect being more evident in propolis samples collected during the rainy season. Under these conditions, the MIC values (in μg/mL) were 32, 64, and 8 for *C. albicans* ATCC 10231, *C. albicans* ATCC 146, and *C. parapsilosis* ATCC 22019, respectively. In contrast, during the dry season, the observed values were 64, >1024, and >1024 for the same strains [68].

A systematic review concluded that the use of propolis caused remission of oral candidiasis and healing caused by the infectious process, both in terms of its effect on the infection and on the inflammatory process, in addition to showing that propolis preparations are heterogeneous and their composition can vary, causing variability in clinical results [69]. Thus, the existence of beneficial effects associated with propolis is evident, despite the variability inherent in a natural product whose production is not fully controlled or standardized. In this context, investigating the influence of seasonality and geographic origin on its biological properties can contribute to a better understanding of these variations, favoring a more targeted exploration of its beneficial effects.

The available scientific evidence on the effects of propolis on *Sporothrix* spp. strains is limited. Fungi of this genus can cause sporotrichosis, which can affect humans and animals and is considered the most important implantation mycosis worldwide [70]. Due to differences in antifungal susceptibility among species, the long treatment period, the high cost, and potential host side effects, among other reasons, alternative therapies are needed to treat *Sporothrix* spp. infections [71]. When the sensitivity of *Sporothrix brasiliensis* strains to brown propolis was tested, all strains were susceptible, including itraconazole-resistant strains, with MIC values ranging from 0.19 to 1.56 mg/mL [72]. In another study, *S. brasilensis* strains exhibited MICs ranging from 0.09 to 0.78 mg/mL [73].

In this study, *Sporothrix* spp. strains are sensitive to all propolis extracts, in varying degrees (Table 6), with *Sporothrix globosa* being the most sensitive. Extracts derived from green and red propolis showed more promising results.

**Figure 3:**
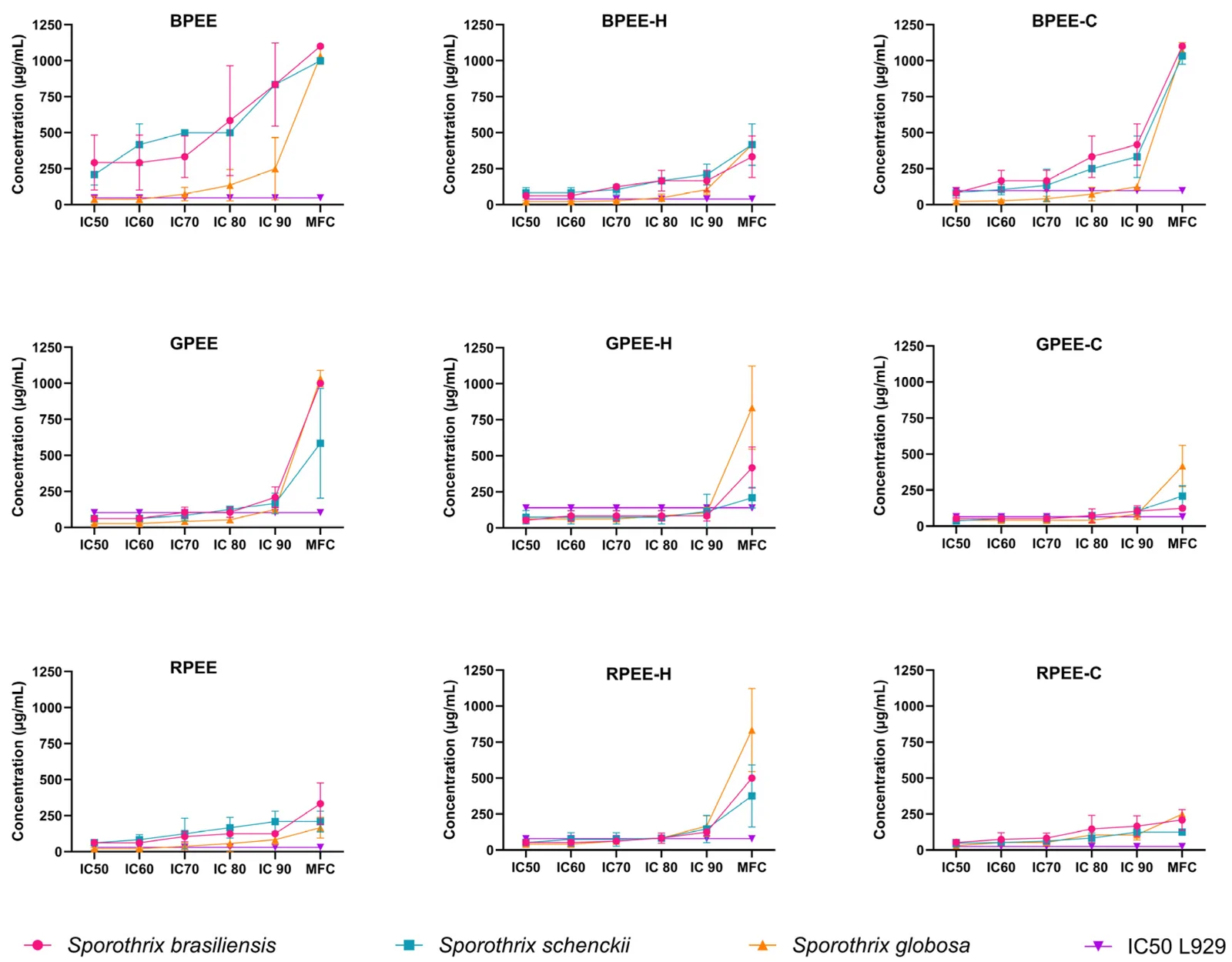
Graphs comparing the concentration (μg/mL) to inhibit 50% (IC50), 60% (IC60), 70% (IC70), 80% (IC80), and 90% (IC90) of the growth of *Sporothrix* spp. species, compared with the average IC50 value of the L929 cell line. Ethanolic Extract of Blue Propolis (BPEE), Ethanolic Extract of Blue Propolis-Hexane Fraction (BPEE-H), Ethanolic Extract of Blue Propolis-Chloroform Fraction (BPEE-C), Ethanolic Extract of Green Propolis (GPEE), Ethanolic Extract of Green Propolis-Hexane Fraction (GPEE-H), Ethanolic Extract of Green Propolis-Chloroform Fraction (GPEE-C), Ethanolic Extract of Red Propolis (RPEE), Ethanolic Extract of Red Propolis-Hexane Fraction (RPEE-H), Ethanolic Extract of Red Propolis-Chloroform Fraction (RPEE-C).

When comparing the IC50 and IC_90_ values of propolis extracts against *Sporothrix* spp., the predominance of IC50 values above the average is observed in the L929 cell line (Figure 3), suggesting that concentrations that inhibit microorganism growth may reflect cellular cytotoxicity.

Therefore, despite its activity against *Sporothrix* spp., its use is not safe because the doses required to inhibit their growth exceed the IC50 concentrations, posing a potential risk. One strategy to explore is determining whether there is a synergistic effect between propolis extracts and the antifungals used to treat these infections.

### 2.6. Possible Role of Ferric Ions in the Blue Coloration of Propolis

**Figure 4:**
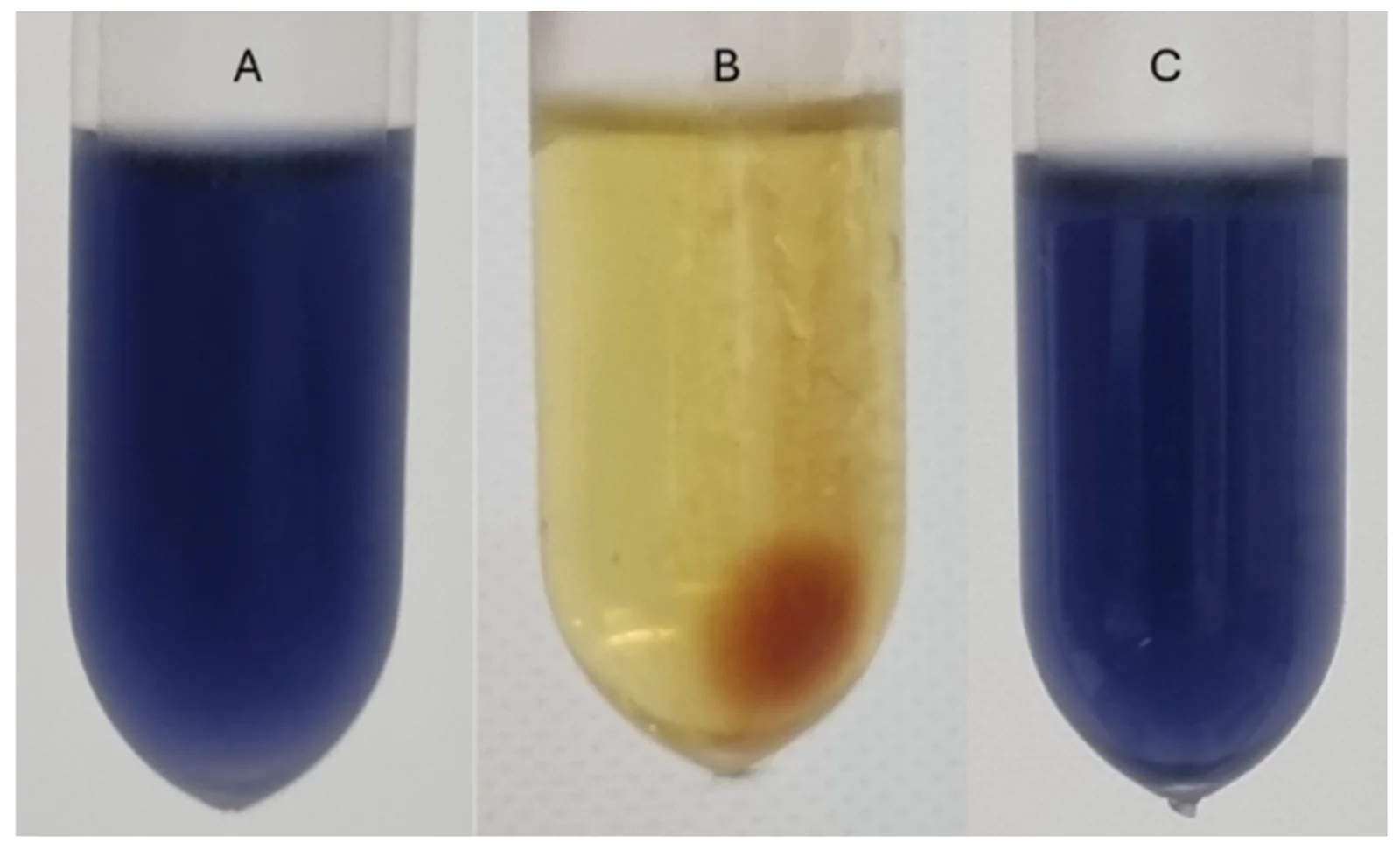
Color changes of the blue propolis solution in its original form (**A**), after NaOH addition (**B**), and after FeCl_3_ addition (**C**).

Initially, the blue propolis solution exhibited its characteristic blue coloration, which disappeared upon addition of NaOH, yielding a yellow solution. Following centrifugation, the formation of a reddish-brown precipitate was observed, suggesting the precipitation of iron species under alkaline conditions. Subsequently, the addition of FeCl_3_ restored the characteristic blue coloration of the propolis solution (Figure 4). This experiment was designed to investigate the possible involvement of phenolic–iron complex formation in the mechanism responsible for the distinctive blue color of this newly identified type of propolis.

The interaction between phenolic compounds and iron has been known for decades and is characterized by the formation of colored complexes upon reaction with ferric chloride [74]. Consistent with the observations obtained in the present study, previous reports have shown that ferric chloride-derived phenolic complexes exhibit an absorption band with a maximum around 558 nm [75] In this context, the presence of an absorption band in the visible region, centered near 600 nm in the blue propolis solution, together with its dependence on the presence of Fe^3+^ ions, suggests that the observed coloration is associated with the formation of iron–phenolic complexes involving compounds present in the propolis extract.

**Figure 5:**
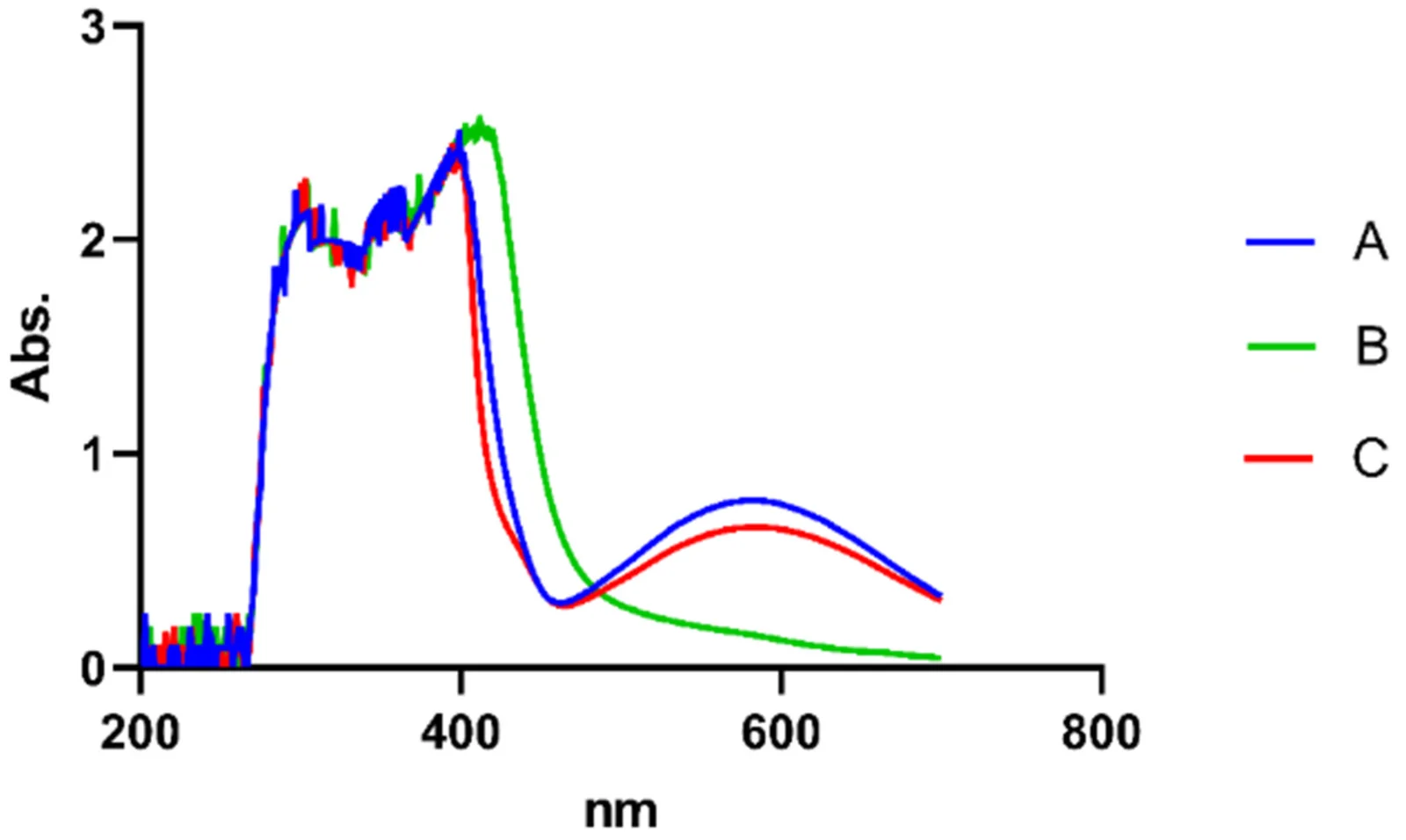
UV–Vis absorption spectra of blue propolis under different experimental conditions: (**A**) untreated propolis solution, (**B**) after NaOH addition followed by centrifugation, and (**C**) after FeCl_3_ addition.

The UV–Vis spectra further supported this hypothesis. The untreated blue propolis solution displayed a distinct absorption band around 600 nm, consistent with its characteristic blue coloration. After NaOH addition, this band disappeared, indicating the loss of the chromophore responsible for the blue color. This effect may be attributed to the precipitation of Fe^3+^ ions as insoluble iron hydroxides under alkaline conditions, thereby disrupting the formation of the iron–phenolic complex. Following the addition of FeCl_3_, the absorption band in the same spectral region was restored, accompanied by the reappearance of the blue coloration (Figure 5). Together, these findings provide preliminary evidence that ferric ions play an important role in the blue coloration of this propolis type, likely through complexation with phenolic constituents.

### 2.7. Limitations and Perspectives

The chemical composition of propolis is directly related to the plant resources available in the vicinity of meliponaries and apiaries, and is influenced by the type of vegetation cover, land use, and the region’s floristic diversity. In this context, a limitation of the present study was the absence of a mapping of the blue propolis collection areas. Environmental characterization, including the identification of the type of vegetation (e.g., native forest fragments, reforestation areas, or agricultural areas), as well as a survey of the main floral resources available, could significantly contribute to understanding the variations in the observed chemical composition and, especially, assist in the identification of compounds in blue propolis. Thus, future studies should prioritize investigating the botanical origin of this new type of propolis, enabling correlation between specific plant species and specific metabolites.

Although no differences were observed in the qualitative composition of the ethanolic extracts and their fractions, differences in biological activity were observed, including the occurrence of synergistic effects in certain cases. This result suggests that the observed activity may be associated with interactions among multiple compounds, rather than with the presence of isolated metabolites. Thus, further approaches can be used to elucidate the compounds responsible for the biological activity. In this sense, the application of bioassay-guided fractionation strategies shows promise, enabling successive fractionations guided by biological assays until active compounds are isolated. However, additional efforts are needed to purify these supposedly bioactive compounds, determine their chemical structures, and evaluate their individual activities.

Furthermore, the potential occurrence of synergistic effects reinforces the need for deeper analytical investigation of propolis extracts. The optimization of chromatographic and mass spectrometric methods may broaden the detection and annotation of compounds not yet identified, especially minor metabolites or constituents not previously reported in the literature that may be relevant to the observed biological activities. In this context, the combination of LC-MS/MS-based profiling, chromatographic purification, NMR-based structural elucidation, and multivariate statistical approaches represents a promising strategy for improving the chemical characterization of blue propolis. These complementary approaches may help identify chemical markers, clarify relationships between chemical composition and biological activity, and support future studies aimed at understanding the mechanisms underlying the bioactivity of this emerging type of propolis.

## 3. Materials and Methods

This project was registered in the National System for the Management of Genetic Heritage (SISGEN), linked to the Ministry of the Environment of the Government of Brazil, under registration number A1C072.

### 3.1. Propolis Samples and Preparation of Extracts

Samples of blue, red, and green propolis were collected from various regions of Brazil and produced by different bee species (Table 7). Meliponiculturists and beekeepers provided the samples, sending them in hermetically sealed packaging. After collection, the samples were stored in a freezer at –20°C (Brastemp, São Paulo, SP, Brazil) until use.

Propolis extracts were prepared by macerating 30.0 g of raw propolis in 300.0 mL of absolute ethanol (Êxodo Científica, Sumaré, SP, Brazil) for 6 h (experimentally determined extraction time) under magnetic stirring at room temperature. Insoluble solids present in the crude propolis extract were removed by centrifugation (1800× *g*, 15 min, 25 °C) (Eppendorf, Hamburg, Germany). The extract was then stored at −20°C for 18 h to promote the precipitation of residual wax, which was subsequently removed by centrifugation (1800× *g*, 15 min, 0 °C). The dewaxed extract was concentrated using a rotary evaporator (Nova Técnica, Piracicaba, SP, Brazil) under reduced pressure at 40 °C until complete solvent removal. After wax removal and solvent evaporation, the resulting extract was designated as the ethanolic extract (EE) and stored at 4 °C.

In addition to extraction with absolute ethanol, the EE was subjected to liquid–liquid partitioning. Briefly, the EE was dissolved in a water:methanol mixture (1:1, *v*/*v*) and sequentially extracted with 50 mL portions of solvents of increasing polarity, namely hexane, chloroform (Êxodo Científica), and ethyl acetate (Neon, Suzano, SP, Brazil). The solvents from the resulting fractions were also removed under reduced pressure at 40 °C using a rotary evaporator.

Stock solutions of the extracts were prepared at 1 mg/mL in methanol for chemical characterization and at 100 mg/mL in dimethylsulfoxide (DMSO) for biological assays (cytotoxicity and antimicrobial activity). Both stock solutions were filtered (0.22 μm membrane) and stored at –20 °C until experimental use.

### 3.2. Chemical Characterization of Propolis Extracts

The chemical characterization of the propolis extracts was performed by high-performance liquid chromatography coupled to mass spectrometry (LC-MS), using an Xevo G3 quadrupole time-of-flight (Q-TOF) hybrid mass spectrometer (Waters Corporation, Milford, MA, USA) equipped with an electrospray ionization (ESI) source, operated in both positive and negative ionization modes. Chromatographic separation was carried out on an Acquity UPLC BEH C18 column (50 mm × 2.1 mm, 1.7 µm). Elution was performed in gradient mode using water as mobile phase A and methanol as mobile phase B, both containing 0.1% (*v*/*v*) formic acid. The flow rate was 0.4 mL min^−1^, the column temperature was maintained at 40 °C, and the injection volume was 1 µL. The elution gradient was programmed as follows: 0–1 min, 80–50% A; 1–7.5 min, 50–20% A; 7.5–9 min, 20–2% A; 9–11 min, 2% A; 11–11.01 min, 2–80% A; and 11.01–12 min, 80% A. Mass spectrometric analysis was performed using capillary voltages of 3.0 kV and 2.5 kV in positive and negative ionization modes, respectively, a cone voltage of 40 V, source temperature of 120 °C, desolvation temperature of 500 °C, desolvation gas flow rate of 900 L h^−1^, and cone gas flow rate of 40 L h^−1^. Nitrogen was used as the desolvation and cone gas, while argon was employed as the collision gas. Leucine enkephalin was continuously infused as the lock mass reference to ensure mass accuracy throughout the analysis. Mass spectra were acquired in the m/z range of 50–1200 using MSE acquisition mode, with a low collision energy of 6 V and a high collision energy ramp from 15 to 45 V. Compounds were tentatively identified based on accurate mass measurements and fragmentation patterns, with a mass error of <5 ppm.

### 3.3. In Vitro Evaluations

The biological activity of blue propolis, including cellular cytotoxicity and antimicrobial activity against bacterial and fungal strains, was evaluated using EE and its fractions (hexane, chloroform, and ethyl acetate).

#### 3.3.1. Assessment of Cellular Cytotoxicity

The cytotoxicity of propolis extracts was evaluated against non-tumor murine fibroblasts (L929, NCTC clone 929; BCRJ code 0188), an adherent fibroblast cell line derived from normal subcutaneous connective tissue of a male C3H/An mouse (*Mus musculus*), obtained from the Rio de Janeiro Cell Bank (BCRJ, Rio de Janeiro, RJ, Brazil), using the tetrazolium salt (3-(4,5-dimethylthiazol-2-yl)-2,5-diphenyltetrazolium bromide) (MTT) colorimetric method [76]. The cell line was cultured in RPMI-1640 medium (Gibco, Grand Island, NY, USA) supplemented with fetal bovine serum (10% *v*/*v*) (Santa Cruz Biotechnology Inc., Santa Cruz, CA, USA) and gentamicin (30 μg/mL) at 37 °C with 5% CO_2_ and approximately 99% relative humidity (COM-170AIC-PE CO2 incubator, PHCbi Europe, Breda, Holanda). The cell line was seeded in 96-well flat-bottomed plates at a cell concentration of 2.0 × 10^3^ cells per well and incubated (37 °C with 5% CO_2_). After 24 h of incubation, the cells were treated with different concentrations (12.5–150 µg/mL) of the extracts and incubated for an additional 72 h. 5-fluorouracil (0.06–4 μM) was used as a positive control, and DMSO (0.3% *v*/*v*) as a negative control. After the incubation period, the medium was removed, and MTT (0.5 mg/mL) (Sigma-Aldrich, St. Louis, MO, USA) was added to the wells. The plates were incubated (37 °C with 5% CO_2_) for 2 h, the MTT solution was removed, and the formazan crystal violet formed was solubilized with DMSO. Absorbance was measured using a microplate reader at 570 nm (Thermo Fischer Scientific, Waltham, MA, USA). Three independent experiments were performed. The readings were compared with the control group for cell growth. The half-maximum inhibitory concentration (IC_50_) values were estimated using GraphPad Prism^®^ software version 8 (GraphPad Software Inc., San Diego, CA, USA), and the results were expressed as IC_50_ and as a percentage of cell viability, calculated using the following formula:

Cell Viability (%) = [(O.D570nmSample − O.D570nmBlank)/(O.D570nmSample − O.D570nmBlank)] × 100(1)

#### 3.3.2. Evaluation of Antibacterial Activity

To evaluate the antibacterial activity of propolis extracts, the Minimum Inhibitory Concentration (MIC) was determined by broth microdilution, according to the Clinical and Laboratory Standards Institute (CLSI) [77]. Eight bacterial strains were used, obtained from the American Type Culture Collection (ATCC), including two Gram-negative strains (*Escherichia coli*, ATCC 25922; *Pseudomonas aeruginosa*, ATCC 27853) and six Gram-positive strains (*Enterococcus faecalis*, ATCC 29212; *Enterococcus faecalis*, ATCC 51299, vancomycin-resistant (VRE) and high-level aminoglycoside-resistant (HLAR) enterococcus; *Enterococcus faecium*, ATCC 6569; *Staphylococcus aureus*, ATCC 6538; *Staphylococcus aureus*, ATCC 33591, methicillin-resistant strain (MRSA); *Staphylococcus epidermidis*, ATCC 12228). The microorganisms were preserved at −20 °C in 20% (*v*/*v*) glycerol. Before use, they were reactivated in Brain Heart Infusion (BHI) (37 °C, 24 h), followed by streaking on Mueller–Hinton agar. With the bacteria grown on the solid medium, the inoculum was prepared by diluting an initial bacterial suspension to 0.5 on the McFarland scale in a 1:20 ratio in saline solution (0.85%, *v*/*v*). In 96-well U-bottom plates, Mueller–Hinton broth and propolis extracts were added in serial dilutions (7.81–1000 μg/mL). Then the inoculum was added, and the plate was incubated (37 °C, 24 h), resulting in a microbial population of 5 × 10^5^ CFU/mL. Gentamicin, vancomycin, oxacillin, and ampicillin were used as positive controls, and DMSO (2%, *v*/*v*) as a negative control. The MIC was determined as the lowest concentration capable of inhibiting microbial growth, determined visually. To determine the Minimum Bactericidal Concentration (MBC), 10 µL of the well corresponding to the MIC and 3 concentrations above it were plated. Three independent experiments were performed.

The ratio of the MBC to the MIC was used to evaluate the antimicrobial activity profile of the extracts. For each sample, the results were interpreted as follows: MBC/MIC values > 4 indicate a bacteriostatic effect, while values ≤ 4 indicate a bactericidal effect.

#### 3.3.3. Evaluation of Antifungal Activity

The inhibitory concentration for fungi was determined by broth microdilution, according to the European Committee on Antimicrobial Susceptibility Testing (EUCAST) protocol [78]. Six fungal strains were used (*Candida albicans*, ATCC 10231; *Candida parapsilosis*, ATCC 22019; *Pichia kudriavzevii* (formerly *Candida krusei*), ATCC 6258; *Sporothrix schenckii*, CFP 00551; *Sporothrix brasiliensis*, CFP 00746; *Sporothrix globosa*, CFP 01021). The fungi were preserved on Sabouraud agar and reactivated by plating on the same medium, in which Candida spp. strains were incubated for 24 h at 35 °C, and *Sporothrix* spp. for 5 days at 25 °C, followed by 7 days at 35 °C. The inoculum was prepared by growing yeast cells on solid medium, then suspending the cells at 0.5 McFarland scale in sterile distilled water, followed by dilution (1:10) in RPMI 1640 medium (supplemented with 2% (*w*/*v*) glucose and MOPS, pH 7.0). The inoculum was added to wells of a 96-well flat-bottomed plate, followed by the addition of propolis at different concentrations prepared in RPMI 1640 medium (7.81–1000 μg/mL). Amphotericin B and itraconazole were used as positive controls, and DMSO (2% *v*/*v*) as a negative control. The plate was then incubated for 72 h at 35 °C. After the incubation period, absorbance was measured using a microplate reader at 405 nm. Using absorbance data, IC_50_, IC_60_, IC_70_, IC_80_, and IC_90_ were calculated as the concentrations that inhibit 50%, 60%, 70%, 80%, and 90% of the growth of the tested microorganisms, respectively, using GraphPad Prism 8.0 software. Three independent experiments were performed. The minimum fungicide concentration (MFC) was determined by plating the contents of wells that showed no visible growth.

#### 3.3.4. Determination of the Selectivity Index

The selectivity index (SI) of the propolis extracts was determined based on the ratio between the IC_50_ values obtained for non-tumor cells (L929) and the minimum inhibitory concentration (MIC) values for bacteria and the inhibitory concentration for fungi. SI values greater than 1 indicate that the extract inhibits the microorganism before causing cytotoxicity, while SI values less than 1 indicate that the extract may cause cytotoxicity before causing inhibition of microbial growth.

Selectivity Index = IC_50_ L929/a (2)

where, IC_50_ L929 is the IC_50_ value obtained for the respective propolis extract in the L929 cell line; “a” may be values of (1) minimum inhibitory concentration for bacteria and (2) inhibitory concentration for fungi.

### 3.4. Investigation of the Participation of Ferric Ions in the Blue Coloration of Propolis

A blue propolis solution was prepared at a concentration of 10 mg/mL in absolute ethanol. Subsequently, 50 µL of 0.5 M NaOH was added to the solution, followed by homogenization and centrifugation at 1800× *g* for 2 min. The supernatant was then collected and treated with 250 µL of a 1% (*w*/*v*) FeCl_3_ solution. Spectrophotometric analyses were performed in the 200–800 nm wavelength range at three stages: (A) the propolis solution without reagent addition, (B) after NaOH addition and centrifugation, and (C) after FeCl_3_ addition. Absolute ethanol was used as a blank for all spectrophotometric measurements.

### 3.5. Statistical Analysis

All values from in vitro biological assays were reported as mean ± standard deviation. Data were analyzed using one-way analysis of variance (ANOVA) with post-hoc multiple comparisons (Tukey’s test) to assess statistical differences, assuming normality, using the Shapiro–Wilk test. Significance was accepted if *p* < 0.05. All analyses were conducted using GraphPad Prism 8.0 software version 8. Statistical analyses were performed for cytotoxicity and antibacterial activity assays. Antifungal activity results were reported descriptively, and no inferential statistical analysis was conducted.

## 4. Conclusions

Under the experimental conditions adopted in this study, blue geopropolis exhibited the highest antibacterial activity against Gram-positive bacteria among the evaluated bee products. Although it was not the least cytotoxic, it demonstrated a superior selectivity index. Antifungal activity against *Candida* spp. and *Pityrosporum kudriavzevii* was limited. In contrast, variable activity was observed against *Sporothrix* spp. strains, mirroring the patterns found for the other propolis types. Nevertheless, this antifungal effect occurred at concentrations higher than the IC_50_ for L929 cells, indicating a low selectivity index for fungal cells.

A major strength of this study is that blue geopropolis, green propolis, and red propolis were evaluated under identical experimental conditions, utilizing the same extraction method, biological assay methodologies, microorganisms, and cell lines. This rigorous standardization minimized methodological variability, allowing a more reliable comparison of their chemical compositions and biological activities than comparisons based solely on literature data. The purpose of this benchmark was not to establish equivalence among bee products of different entomological origins, but rather to map the distinct similarities and differences in their chemical profiles and biological properties.

It must be emphasized that the chemical composition of both propolis and geopropolis is fundamentally influenced by factors such as botanical origin, bee species, geographic location, and seasonality. Therefore, the findings of this study should be interpreted within the specific context of the analyzed samples; broader comparative investigations encompassing different production regions and collection periods are necessary before generalizing the biological performance of these bee products.

Overall, these results indicate that blue geopropolis possesses a distinctive chemical profile and promising biological activity, particularly against Gram-positive bacteria, supporting its potential as a valuable source of bioactive compounds for future biotechnological and pharmacological applications.

## Figures and Tables

**Table 1:** Compounds identified from ethanolic extracts of propolis samples (blue, green and red) and their hexane and chloroform fractions using ultraperformance liquid chromatography (UPLC)/quadrupole time-of-flight (qTOF) with electrospray ionization (ESI) in positive and negative modes.

N.	Compound	Molecular Formula	Adduct	*m*/*z*	Error (ppm)	Fragments	BPEE	BPEE-H	BPEE-C	GPEE	GPEE-H	GPEE-C	RPEE	RPEE-H	RPEE-C	References
Phenolics																
1	Coumarin	C_9_H_6_O_2_	[M+H]^+^	147.0434	−4.76	91	ND	ND	ND	P	ND	P	ND	ND	ND	[21]
2	Drupanin (3-prenyl-4-hydroxycinnamic acid)	C_14_H_16_O_3_	[M-H]^−^	231.1018	−3.65	132; 187; 231	P	P	ND	P	P	P	ND	ND	ND	[22]
3	2’-Hydroxy-4’-methoxychalcone	C_16_H_14_O_3_	[M-H]^−^	253.0862	−3.27	237; 253; 136; 161	ND	ND	ND	ND	ND	ND	P	P	P	[23]
4	Liquiritigenin	C_15_H_12_O_4_	[M-H]^−^	255.0656	−2.65	119; 135; 120; 255	ND	ND	ND	ND	ND	ND	P	P	P	[23]
5	Liquiritigenin or Isoliquiritigenin	C_15_H_12_O_4_	[M-H]^−^	255.0657	0.07	91; 119; 135; 255	P	P	P	P	P	P	P	P	P	[24]
6	Demethylmedicarpin	C_15_H_12_O_4_	[M-H]^−^	255.0657	−2.21	151; 213; 255	P	P	P	P	P	P	P	P	P	[23]
7	Pinocembrine	C_15_H_12_O_4_	[M-H]^−^	255.0660	−1.10	145; 151; 211; 213	P	P	P	P	P	P	P	P	P	[25]
8	Formononetin	C_16_H_12_O_4_	[M-H]^−^	267.0664	0.40	267; 252; 223;	P	P	P	P	P	P	P	P	P	[22]
9	Pinostrobin	C_16_H_14_O_4_	[M-H]^−^	269.0813	−2.46	254	ND	ND	ND	P	P	P	P	P	P	[25,29]
			[M+H]^+^	271.0956	−3.26	131; 167	ND	ND	ND	ND	ND	ND	P	P	P	
10	Galangin	C_15_H_10_O_5_	[M+H]^+^	271.0593	−2.95	131; 165; 197;	ND	ND	ND	P	ND	P	ND	ND	ND	[29]
11	Genistein	C_15_H_10_O_5_	[M+H]^+^	271.0593	−2.95		ND	ND	ND	P	ND	P	P	ND	P	-
12	Naringenin	C_15_H_12_O_5_	[M-H]^−^	271.0600	−4.31	107; 119; 151; 177	ND	ND	ND	ND	ND	ND	P	P	P	[22]
13	2’,6’-Dihydroxy-4’-methoxydihydrochalcone	C_16_H_16_O_4_	[M-H]^−^	271.0979	1.07	109; 135; 254	P	ND	P	P	P	P	P	P	P	[23]
14	Vestitol	C_16_H_16_O_4_	[M-H]^−^	271.0982	2.33	109; 135; 147; 149; 271; 256	P	P	P	P	ND	P	P	P	P	[23]
15	Biochanin A	C_16_H_12_O_5_	[M-H]^−^	283.0604	−2.79	211; 239; 268; 283	ND	ND	ND	P	P	P	P	P	P	[22]
16	Methoxychrysin	C_16_H_12_O_5_	[M-H]^−^	283.0605	−2.63	211; 239; 268	P	ND	ND	ND	ND	P	P	P	P	[25]
17	2-Hydroxynaringenin	C_15_H_12_O_6_	[M-H]^−^	287.0551	−3.60	125; 151; 177; 201; 259; 269	ND	ND	ND	P	P	P	P	ND	P	[27]
18	4’-Hydroxy-3,4,5- trimethoxystilbene	C_17_H_18_O_4_	[M+H]^+^	287.1273	−1.74	225; 241	ND	ND	ND	ND	ND	ND	P	P	P	[21]
19	6-gingerol	C_17_H_26_O_4_	[M-H]^−^	293.1750	−3.00	236; 221	P	P	P	P	P	P	P	P	P	[31]
20	Hispidulin	C_16_H_12_O_6_	[M-H]^−^	299.0552	−3.05	227; 255; 284	ND	ND	ND	P	P	P	P	P	P	[23]
21	Kaempferide	C_16_H_12_O_6_	[M-H]^−^	299.0554	−2.35	151; 284; 299	P	ND	ND	P	P	P	P	P	P	[22]
22	Sativanone	C_17_H_16_O_5_	[M-H]^−^	299.0913	−4.08	225; 269; 284	ND	ND	ND	P	P	P	P	P	P	[21]
23	Artepillin C (3,5-diprenyl-4- hydroxycinnamic acid)	C_19_H_24_O_3_	[M-H]^−^	299.1647	−1.75	299; 255; 200	P	ND	ND	P	P	P	P	ND	P	[22]
24	7-O-methyl aromadendrin	C_16_H_14_O_6_	[M+H]^+^	303.0852	−3.30	229; 257	ND	ND	ND	ND	ND	ND	P	ND	P	[27]
25	Quercetin-3-methyl ether	C_16_H_12_O_7_	[M-H]^−^	315.0508	−0.87	151; 271; 300	P	ND	ND	P	P	P	P	P	P	[30]
26	Rhamnetin	C_16_H_12_O_7_	[M-H]^−^	315.0508	−0.87	165; 300	P	ND	ND	P	P	P	P	P	P	[25]
27	5,6-Dihydroxy-3’,4’-dimethoxyflavanone	C_17_H_16_O_6_	[M-H]^−^	315.0865	−2.97	121; 151; 315	P	ND	ND	P	ND	P	P	P	P	[23]
28	Violanone	C_17_H_16_O_6_	[M-H]^−^	315.0866	−2.43	135; 285	ND	ND	ND	P	P	P	P	P	P	[21]
29	4-hydroxy-3(E)-(4-hydroxy-3-methyl-2-butenyl)-5-prenyl cinnamic acid	C_19_H_24_O_4_	[M-H]^−^	315.1597	−1.65	186; 198; 241; 253; 271; 285; 297; 315	P	P	P	P	P	P	P	P	P	[22]
30	Betuletol	C_17_H_14_O_7_	[M-H]^−^	329.0656	−3.34	255; 271; 299; 314; 329	P	ND	ND	P	P	P	P	P	P	[22]
31	Viscosine	C_17_H_14_O_7_	[M-H]^−^	329.0656	−3.19	244; 271; 299; 314	ND	ND	ND	P	P	P	P	P	P	[30]
32	Quercetin-3,7-dimethyl ether	C_17_H_14_O_7_	[M-H]^−^	329.0657	−2.89	271; 299; 314	P	ND	ND	P	P	P	P	ND	P	[28,29]
		C_17_H_14_O_7_	[M+H]^+^	331.0804	−2.50	137; 151; 301; 316	ND	ND	ND	P	ND	P	ND	ND	ND	
33	Artepillin C derivative	C_20_H_26_O_4_	[M-H]^−^	329.1750	−2.37	255; 299	ND	ND	ND	P	P	P	ND	ND	ND	[26]
34	Anacardic acid (15:1)	C_22_H_34_O_3_	[M-H]^−^	345.2431	−1.16	301; 345	P	ND	ND	P	P	P	P	P	P	[22]
35	Anacardic acid (15:0)	C_22_H_36_O_3_	[M-H]^−^	347.2587	−1.32	303; 347	P	ND	ND	P	P	P	P	P	P	[22]
36	Baccharin (3-prenyl-4-(dihydrocinnamoyloxy) cinnamic acid)	C_23_H_24_O_4_	[M-H]^−^	363.1596	−1.66	149; 187	P	P	P	P	P	P	P	ND	P	[22]
37	Anacardic acid (17:2)	C_24_H_36_O_3_	[M-H]^−^	371.2589	−0.55	327; 371	P	P	P	P	P	P	P	P	P	[22]
38	Anacardic acid (17:1)	C_24_H_38_O_3_	[M-H]^−^	373.2744	−1−21	329; 373	P	P	P	P	P	P	P	P	P	[22]
39	Mepuberin	C_23_H_26_O_5_	[M+H]^+^	383.1853	0.26	299; 313; 327; 355	P	P	P	ND	ND	ND	P	P	P	[27]
40	Dicoumaric prenyl ester	C_23_H_22_O_6_	[M-H]^−^	393.1336	−1.95	119; 145; 163	P	P	P	P	P	P	P	ND	ND	[22]
41	Unidentified anacardic acid	C_26_H_42_O_3_	[M-H]^−^	401.3062	0.16	357; 401	P	P	P	P	P	P	P	P	P	[22]
42	(E)-3-{4-hydroxy-3-[(E)-4-(2,3- dihydrocinnamoyloxy)-3-methyl-2- butenyl]-5-prenyl-phenyl}-2- propenoic acid	C_28_H_32_O_5_	[M-H]^−^	447.2173	−0.98	149; 197; 297	P	P	P	P	P	P	P	P	P	[22]
43	Xanthochymol (Guttiferone E)	C_38_H_50_O_6_	[M-H]^−^	601.3546	1.95	108; 109; 177; 202	P	P	P	P	P	P	P	P	P	[23]
44	Artepillin derivative	C_38_H_46_O_7_	[M-H]^−^	613.3170	−0.18	281; 299; 613	P	P	P	P	P	P	P	P	P	[22]
Terpenoids																
45	Pinusenocarp	C_17_H_24_O_4_	[M-H]^−^	291.1590	−4.14	227; 229; 273	P	P	P	ND	ND	ND	ND	ND	ND	[27]
46	Dehydroabietic acid	C_20_H_28_O_2_	[M-H]^−^	299.2011	−1.93	299	P	P	P	P	P	P	P	P	P	[32]
47	Pimaric/isopimaric acid	C_20_H_30_O_2_	[M+H]^+^	303.2318	−0.19	257; 201	P	P	P	ND	ND	ND	ND	ND	ND	[29]
48	7-oxydehydroabietic acid	C_20_H_26_O_3_	[M+H]^+^	315.1955	−2.13	297	P	P	P	P	P	P	ND	ND	ND	[27]
49	Cupressic acid	C_20_H_32_O_3_	[M-H]^−^	319.2271	−2.52	319	P	P	P	ND	ND	ND	ND	ND	ND	[22]
50	Agathic acid	C_20_H_30_O_4_	[M-H]^−^	333.2061	−3.16	333	P	P	P	ND	ND	ND	ND	ND	ND	[22]
51	Junicedric acid (dihydroagathic acid)	C_20_H_32_O_4_	[M-H]^−^	335.2216	−3.43	335	P	P	P	ND	ND	ND	ND	ND	ND	[22]
52	Mangiferonic acid	C_30_H_46_O_3_	[M-H]^−^	453,.3370	−0.89	453	P	P	P	P	P	P	P	P	P	[22]
53	Mangiferolic or isomangiferolic acid	C_30_H_48_O_3_	[M-H]^−^	455.3525	−1.18	455	P	P	P	P	P	P	P	P	P	[22]
54	Unidentified triterpene	C_30_H_46_O_4_	[M-H]^−^	469.3320	−0.76	407; 439; 451; 469	P	P	P	P	P	P	P	P	P	[22]
Lipids																
55	Palmitic acid	C_16_H_32_O_2_	[M-H]^−^	255.2323	−2.72	255	P	P	P	P	P	P	P	P	P	[22]
56	Oleic acid	C_18_H_34_O_2_	[M-H]^−^	281.2478	−3.02	281	P	P	P	P	P	P	P	P	P	[22]
57	Stearic acid	C_18_H_36_O_2_	[M-H]^−^	283.2637	−2.04	283	P	P	P	P	P	P	P	P	P	[22]
Carbohydrate																
58	Melibiose	C_12_H_22_O_11_	[M-HCOO]^−^	387.1137	0.94	179; 221	P	P	P	P	P	P	P	P	P	[31]
Other																
59	Unidentified compound	C_32_H_46_O_6_	[M+H]^+^	527.3388	3.91	275; 317; 335; 357; 399	P	P	P	P	ND	P	P	P	ND	[27]

(P) Present, (ND) Not detected. Ethanolic Extract of Blue Propolis (BPEE), Ethanolic Extract of Blue Propolis-Hexane Fraction (BPEE-H), Ethanolic Extract of Blue Propolis-Chloroform Fraction (BPEE-C), Ethanolic Extract of Green Propolis (GPEE), Ethanolic Extract of Green Propolis-Hexane Fraction (GPEE-H), Ethanolic Extract of Green Propolis-Chloroform Fraction (GPEE-C), Ethanolic Extract of Red Propolis (RPEE), Ethanolic Extract of Red Propolis-Hexane Fraction (RPEE-H), Ethanolic Extract of Red Propolis-Chloroform Fraction (RPEE-C).

**Table 2:** IC_50_ values (range and mean ± SD) for the cytotoxicity of propolis ethanolic extracts and their fractions in L929 cells (μg/mL).

Extract	IC_50_ Mean ± SD (μg/mL)
BPEE	47 ± 4 ^ab^
BPEE-H	39 ± 2 ^bd^
BPEE-C	101 ± 12 ^c^
GPEE	101 ± 8 ^c^
GPEE-H	138 ± 17 ^f^
GPEE-C	69 ± 10 ^ae^
RPEE	29.8 ± 1.3 ^bd^
RPEE-H	89 ± 16 ^ce^
RPEE-C	25 ± 0.6 ^d^

Statistical differences were evaluated by one-way ANOVA followed by Tukey’s post hoc test. ^abcdef^ Values in the same column followed by the same letter do not differ statistically (*p* > 0.05). Ethanolic Extract of Blue Propolis (BPEE), Ethanolic Extract of Blue Propolis-Hexane Fraction (BPEE-H), Ethanolic Extract of Blue Propolis-Chloroform Fraction (BPEE-C), Ethanolic Extract of Green Propolis (GPEE), Ethanolic Extract of Green Propolis-Hexane Fraction (GPEE-H), Ethanolic Extract of Green Propolis-Chloroform Fraction (GPEE-C), Ethanolic Extract of Red Propolis (RPEE), Ethanolic Extract of Red Propolis-Hexane Fraction (RPEE-H), Ethanolic Extract of Red Propolis-Chloroform Fraction (RPEE-C).

**Table 3:** Antibacterial susceptibility of *Staphylococcus* spp. to propolis extracts.

Extracts	Microorganisms											
	*S. aureus* ATCC6538				*S. aureus* ATCC33591				*S. epidermidis* ATCC12228			
	MIC	MBC	MBC/MIC ^a^	IS	MIC	MBC	MBC/MIC ^a^	IS	MIC	MBC	MBC/MIC ^a^	IS
BPEE	36.5	>250	>6.85	1.30	20.8	>125	>6.00	2.28	31.3	>500	>15.97	1.52
BPEE-H	36.5	>250	>6.85	1.06	41.7	>250	>5.99	0.93	41.7	>500	>11.99	0.93
BPEE-C	83.3	>500	>6.00	1.21	114.6	>500	>4.36	0.87	83.3	>1000	>12.00	1.21
GPEE	500	>1000	>2.00	0.20	416.7	1000	2.40	0.24	666.7	>1000	>1.50	0.15
GPEE-H	>1000	>1000	>1	ND	1000	ND	ND	0.18	>1000	ND	ND	ND
GPEE-C	250	500	2	0.28	250	500	2	0.28	500.0	>1000	>2	0.14
RPEE	145.8	>1000	>6.86	0.20	208.3	1000	4.80	0.14	166.7	1000	6.00	0.01
RPEE-H	1000	>1000	>1	0.09	500	1000	2	0.18	833.3	>1000	>1.20	0.11
RPEE-C	125	1000	8	0.20	83.3	500	6.00	0.30	375.0	500	1.33	0.07
GEN ^b^	0.58	-	-	-	0.83	-	-	-	0.25	-	-	-
VAN ^b^	0.83	-	-	-	1.67	-	-	-	0.83	-	-	-
OXA ^b^	0.25	-	-	-	256	-	-	-	0.25	-	-	-

^a^ Indicates bacteriostatic (MBC/MIC > 4) or bactericidal (MBC/MIC < 4) properties. IS = Selectivity Index; ^b^ Positive control, GEN = Gentamicin; VAN = Vancomycin; OXA = Oxacillin. Ethanolic Extract of Blue Propolis (BPEE), Ethanolic Extract of Blue Propolis-Hexane Fraction (BPEE-H), Ethanolic Extract of Blue Propolis-Chloroform Fraction (BPEE-C), Ethanolic Extract of Green Propolis (GPEE), Ethanolic Extract of Green Propolis-Hexane Fraction (GPEE-H), Ethanolic Extract of Green Propolis-Chloroform Fraction (GPEE-C), Ethanolic Extract of Red Propolis (RPEE), Ethanolic Extract of Red Propolis-Hexane Fraction (RPEE-H), Ethanolic Extract of Red Propolis-Chloroform Fraction (RPEE-C). ND: not determined.

**Table 4:** Antibacterial susceptibility of *Enterococcus* spp. to propolis extracts.

Extracts	Microorganisms											
	*E. faecalis* ATCC29212				*E. faecalis* ATCC51299				*E. faecium* ATCC6569			
	MIC	MBC	MBC/MIC ^a^	SI	MIC	MBC	MBC/MIC ^a^	SI	MIC	MBC	MBC/MIC ^a^	SI
BPEE	62.50	>500	>8.06	0.76	52.08	250	4.80	0.91	145.83	>1000	>6.86	0.32
BPEE-H	125.00	>1000	>8.00	0.311	83.33	500	6.00	0.47	166.67	>1000	>6.00	2.33
BPEE-C	250.00	>500	>2.00	0.40	166.67	500	3.00	0.60	500.00	>1000	>2.00	0.20
GPEE	666.67	>1000	>1.50	0.15	1000.0	ND	ND	0.10	1000	ND	ND	0.10
GPEE-H	>1000	ND	ND	ND	>1000	ND	ND	ND	>1000	ND	ND	ND
GPEE-C	666.67	>1000	>1.50	0.10	416.6	>1000	>2.40	0.17	666.67	>1000	>1.50	0.10
RPEE	333.33	1000	>3.00	0.09	291.67	1000	3.43	0.10	416.67	>1000	>2.40	0.07
RPEE-H	>1000	ND	ND	ND	>1000	ND	ND	ND	>1000	ND	ND	ND
RPEE-C	416.67	1000	>2.40	0.08	250	>1000	>4	0.10	583.3	>1000	>1.71	0.04
GEN ^b^	10.67	-	-	-	>2000	-	-	-	13.33	-	-	-
VAN ^b^	1	-	-	-	170.66	-	-	-	0.67	-	-	-

^a^ Indicates bacteriostatic (MBC/MIC > 4) or bactericidal (MBC/MIC < 4) properties. SI = Selectivity Index; ^b^ Positive control, GEN = Gentamicin; VAN = Vancomycin. Ethanolic Extract of Blue Propolis (BPEE), Ethanolic Extract of Blue Propolis-Hexane Fraction (BPEE-H), Ethanolic Extract of Blue Propolis-Chloroform Fraction (BPEE-C), Ethanolic Extract of Green Propolis (GPEE), Ethanolic Extract of Green Propolis-Hexane Fraction (GPEE-H), Ethanolic Extract of Green Propolis-Chloroform Fraction (GPEE-C), Ethanolic Extract of Red Propolis (RPEE), Ethanolic Extract of Red Propolis-Hexane Fraction (RPEE-H), Ethanolic Extract of Red Propolis-Chloroform Fraction (RPEE-C). ND: not determined.

**Table 5:** In vitro antifungal susceptibility of *Candida* spp. and *Pichia kudriavzevii* to propolis extracts, itraconazole, and amphotericin B.

Extracts	Microorganisms								
	*C. albicans* ATCC 10231			*C. parapsilosis ATCC 22019*			*P. kudriavzevii* ATCC 6258		
	IC_50_	IC_90_	MFC	IC_50_	IC_90_	MFC	IC_50_	IC_90_	MFC
BPEE	>1000	>1000	>1000	>1000	>1000	>1000	>1000	>1000	>1000
BPEE-H	>1000	>1000	>1000	1000	>1000	>1000	250	500	>1000
BPEE-C	>1000	>1000	>1000	>1000	>1000	>1000	>1000	>1000	>1000
GPEE	1000	>1000	>1000	>1000	>1000	>1000	500	>1000	>1000
GPEE-H	500	1000	1000	250	500	500	125	125	250
GPEE-C	1000	>1000	>1000	500	>1000	>1000	250	250	500
RPEE	250	1000	1000	500	1000	1000	250	1000	1000
RPEE-H	1000	1000	1000	250	500	1000	125	250	250
RPEE-C	500	1000	1000	500	>1000	>1000	500	1000	1000
Itraconazole ^a^	0.125	0.5	ND	0.125	0.5	ND	0.125	0.5	ND
Anfotericin B ^a^	0.25	0.25	ND	0.25	0.5	ND	1.00	1.00	ND

^a^ Positive control; ND: Not detected. Ethanolic Extract of Blue Propolis (BPEE), Ethanolic Extract of Blue Propolis-Hexane Fraction (BPEE-H), Ethanolic Extract of Blue Propolis-Chloroform Fraction (BPEE-C), Ethanolic Extract of Green Propolis (GPEE), Ethanolic Extract of Green Propolis-Hexane Fraction (GPEE-H), Ethanolic Extract of Green Propolis-Chloroform Fraction (GPEE-C), Ethanolic Extract of Red Propolis (RPEE), Ethanolic Extract of Red Propolis-Hexane Fraction (RPEE-H), Ethanolic Extract of Red Propolis-Chloroform Fraction (RPEE-C). IC_50_ and IC_90_: Inhibitory concentration capable of inhibiting 50% and 90% of microorganism growth, respectively.

**Table 6:** In vitro antifungal susceptibility of *Sporothrix* spp. to propolis extracts, itraconazole, and amphotericin B.

Extracts	Microorganisms								
	*S. schenckii* CFP 00551			*S. brasiliensis* CFP 00746			*S. globosa* CFP 01021		
	IC_50_	IC_90_	MFC	IC_50_	IC_90_	MFC	IC_50_	IC_90_	MFC
BPEE	208.33	833.33	1000	291.67	833.33	>1000	36.46	250.00	1000
BPEE-H	83.33	208.33	500	62.50	166.67	250	20.83	104.17	500
BPEE-C	83.33	333.33	1000	83.33	416.67	>1000	20.83	125.00	>1000
GPEE	62.50	166.67	500	62.50	208.33	1000	26.04	125.00	1000
GPEE-H	72.92	114.58	250	52.083	83.33	500	62.50	104.17	1000
GPEE-C	36.46	104.17	250	52.08	104.17	125	41.67	83.33	500
RPEE	62.33	208.33	250	62.50	125.00	250	20.83	83.33	125
RPEE-H	52.08	145.83	500	52.08	125.00	500	41.67	166.67	1000
RPEE-C	46.87	125.00	125	52.08	166.66	250	31.25	104.17	250
Itraconazol ^a^	1.0	2.00	ND	0.83	1.00	ND	0.67	6.67	ND
Anfotericina B ^a^	1.33	2.00	ND	1.67	2.00	ND	0.42	2.67	ND

^a^ Positive control; ND: Not detected. Ethanolic Extract of Blue Propolis (BPEE), Ethanolic Extract of Blue Propolis-Hexane Fraction (BPEE-H), Ethanolic Extract of Blue Propolis-Chloroform Fraction (BPEE-C), Ethanolic Extract of Green Propolis (GPEE), Ethanolic Extract of Green Propolis-Hexane Fraction (GPEE-H), Ethanolic Extract of Green Propolis-Chloroform Fraction (GPEE-C), Ethanolic Extract of Red Propolis (RPEE), Ethanolic Extract of Red Propolis-Hexane Fraction (RPEE-H), Ethanolic Extract of Red Propolis-Chloroform Fraction (RPEE-C). IC_50_ and IC_90_: Inhibitory concentration capable of inhibiting 50% and 90% of microorganism growth, respectively.

**Table 7:** Information on blue, green, and red propolis samples, including bee species, collection site, and geographic coordinates.

Propolis	Bee Species	Collection Site	Geographic Coordinates
Blue	*Melipona quadrifasciata quadrifasciata*	Paranaguá, Paraná, Brazil	25°41′35.22″ S, 48°34′57.05″ W
Green	*Apis mellifera*	Santa Luzia de Mantenópolis, Espírito Santo, Brazil	18°55′47.0″ S, 41°00′08.5″ W
Red	*Apis mellifera*	Marechal Deodoro, Alagoas, Brazil	9°42′34.4″ S, 35°54′7.62″ W

## Data Availability

The original contributions presented in the study are included in the article. Further inquiries can be directed to the corresponding authors.
